# The clinical significance and prognostic implication of autophagy-related gene 13 in human gastric cancer

**DOI:** 10.3389/fonc.2026.1729996

**Published:** 2026-02-06

**Authors:** Yanjie You, Wenmei Li, Yudi Feng, Ling Gao, Tiantian Li, Xiaoli Zhang, Xiaomei Luo

**Affiliations:** 1 Department of Gastroenterology, People’s Hospital of Ningxia Hui Autonomous Region, Ningxia Medical University, Yinchuan, China; 2 Department of Blood Transfusion, People’s Hospital of Ningxia Hui Autonomous Region, Ningxia Medical University, Yinchuan, China

**Keywords:** autophagy-related gene 13, data mining, gastric cancer, immunohistochemistry, survival outcome

## Abstract

**Objective:**

Gastric cancer remains a leading cause of cancer-related death worldwide, underscoring the need for novel prognostic biomarkers to guide personalized therapy. Previous studies have identified that the autophagy-related gene 13 (ATG13) plays an essential role in cell biological processes, while its clinical significance and prognostic values in gastric cancer remain unclear.

**Methods:**

Bioinformatic analyses were conducted to assess the transcription levels and genomic alterations of the ATG13 gene in gastric cancer. Immunohistochemistry was also utilized to evaluate the association between ZHX3 protein expression and clinicopathologic variables as well as patient survival. In addition, cell proliferation, colony formation and invasion assays were performed to examine the impacts of silencing ATG13 expression on gastric cancer cell growth and metastasis.

**Results:**

ATG13 expression was significantly elevated in gastric cancer tissues compared to noncancerous tissues, which was strongly associated with large tumor size and poor outcomes in patients with gastric cancer. Multivariate analysis indicated that ATG13 expression was an independent prognostic indicator for patient survival. Silencing ATG13 expression functionally inhibited growth and metastasis of gastric cancer cells.

**Conclusion:**

The above findings suggest that dysregulation of ATG13 expression is involved in gastric cancer progression and may serve as a candidate survival biomarker for this malignancy, which warrant further validation by further *in vitro*/*in vivo* models and clinical studies to strengthen the mechanistic evidence and translational potential in our future work.

## Introduction

As one of the most common malignancies, gastric cancer has a fifth highest incidence and fourth highest mortality worldwide (1, 2). The initiation of gastric cancer is complex and includes a variety of genetic and environmental factors, such as genetic susceptibility, alcohol and tobacco administration, unsuitable diet and Helicobacter pylori infection (2). Despite numerous advances in diagnosis and surgery curative therapy in recent years, the prognosis of gastric cancer patients remains unsatisfactory (2). It is urgent to identify precise molecular mechanisms and robust novel biomarkers underlying the initiation and progression of gastric cancer to improve the survival outcome.

Autophagy is one conserved catabolic cellular process by which cells degrade long-lived and faulty intracellular components into lysosomes, and its disorder is associated with multiple human diseases including cancer (3). The molecular machinery of autophagy was largely referred to as autophagy-related genes (ATG), among which ATG13 plays a crucial role in the regulation of autophagy by promoting the formation of autophagosomes. ATG13 was originally identified in yeast as a constitutively expressed protein that was genetically linked to ATG1, a protein kinase required for autophagy (3). Mammalian ATG13 forms a complex with the ATG1 homologues ULK1/2, along with FIP200, which localizes to autophagic isolation membranes and regulates autophagosome biogenesis (4, 5). mTOR phosphorylates both Atg13 and ULK1, suppressing ULK1 kinase activity and autophagy (6). ATG13 expression has been reported to be significantly reduced in endometria from women with polycystic ovary syndrome, but increased in epithelial ovarian cancer tissues (7, 8). In addition, ATG13 expression was also been found to be decreased when breast cancer cells treated with cisplatin (9). Although the impact of ATG13 on multiple cancer types is still controversial, i.e., both tumor-suppressor and oncogene functions in different contexts, it has been postulated that ATG13 has the potential to be a valuable therapeutic target or biomarker (10–12). Regarding gastric cancer, it has been found that ATG13 expression is involved in disease progression and associated with patient survival (13–15). However, its expression pattern, clinical relevance, and prognostic value in gastric cancer have not been systematically explored.

To address this gap, we conducted an integrated clinical-translational study. We combined bioinformatic analyses of public cohorts, immunohistochemical validation in a well-characterized patient cohort, and *in vitro* functional experiments. Our objectives were: (1) to determine ATG13 expression in gastric cancer and its association with clinicopathological parameters; (2) to evaluate its value as a prognostic/exploratory biomarker for patient survival; and (3) to explore its pilot nature on gastric cancer cell growth and metastasis.

## Materials and methods

### Study design and patient cohorts

This retrospective study was designed to investigate the clinical significance of ATG13 in gastric cancer. A dual-strategy approach was employed: initial bioinformatics analysis of publicly available datasets, followed by validation in an independent, well-characterized clinical cohort supplemented by functional experiments.

For the bioinformatic cohort, transcriptomic and genomic data were sourced from public repositories including Cancer Genome Atlas (TCGA, https://portal.gdc.cancer.gov) and Genotype-Tissue Expression (GTEx, https://www.genome.gov) projects and providing a large sample size for exploratory analysis (16, 17).

The clinical validation cohort consisted of a tissue microarray (catalog no. HStmA180Su20; Outdo Biotech., Shanghai, China) constructed from formalin-fixed, paraffin-embedded tumor specimens of 99 patients diagnosed with primary stomach adenocarcinoma (STAD). These patients underwent curative resection between January 2011 and December 2012. Additionally, 71 paired adjacent non-cancerous tissue samples were included as controls. Among 99 patients, one case lost the follow-up information and was not included for the survival analysis. The median follow-up period for the cohort of 98 patients was 40 months (range, 2–71 months) from the date of surgery. During the follow-up period, 41.8% (41/98) of patients had died because of disease recurrence and distant metastasis. For survival curve analysis, the percentage of censored cases was 58.2% (57/98). Key inclusion criteria were: (a) histologically confirmed primary STAD, (b) availability of complete clinicopathological and follow-up data, and (c) no prior radiotherapy or chemotherapy before surgery. Exclusion criteria included: (a) incomplete medical records, (b) presence of other synchronous malignancies, and (c) perioperative mortality. Tumor staging was performed according to the 7th edition UICC/AJCC TNM classification system. Tumor grade and stage were classified in accordance with the Union of International Cancer Control (UICC)/American Joint Committee on Cancer (AJCC) pathologic tumor−node−metastasis (TNM) classification, 7th edition (2010). No patients in the study had undergone preoperative radiotherapy or chemotherapy.

### Gene expression profiling interactive analysis database analysis

GEPIA (http://gepia.cancer-pku.cn) is a newly developed interactive web server for analyzing the RNA sequencing expression data of 9, 736 tumors and 8, 587 normal samples from TCGA and GTEx projects (18). GEPIA provides customizable functions such as tumor/normal differential expression analysis according to cancer types or pathological stages, patient survival analysis, similar gene detection, correlation analysis and dimensionality reduction analysis. In the present study, differential transcriptional expression of ATG13 between gastric cancer tissues and normal tissues was assessed via GEPIA database analysis.

### cBioPortal cancer genomics database analysis

The effect of genomic alterations of the ATG13 gene including mutations, deletion and copy-number variance on overall survival (OS), disease−free survival (DFS), disease−specific survival (DSS) and progress−free survival (PFS) of cancer patients were calculated using the cBioPortal online database (http://www.cbioportal.org) (19). The cBioPortal for Cancer Genomics provides visualization, analysis and download of large-scale cancer genomics datasets. An OncoPrint graphic from cBioPortal were utilized to show the proportion and distribution of samples with genomic alterations.

### Immunohistochemistry and evaluation

Immunohistochemical staining was performed using a standard EnVision complex method previously described (20). Briefly, 4-μm sections cut from the tissue microarray chip containing the tissue samples that had been fixed in 10% (vol/vol) neutral-buffered formalin and embedded in paraffin were deparaffinized, rehydrated and incubated with a ATG13 rabbit polyclonal antibody (catalog no. ab105392; Abcam, Cambridge, MA, USA) at a dilution of 1:200 overnight at 4°C, followed by incubation with an Envision antibody complex (anti-mouse/rabbit) using an Envision™ Detection kit (Gene Tech., Shanghai, China). Staining was visualized by incubation with 3, 3’-diaminobenzidine (DAB) as the chromogen substrate, resulting in a brown-colored precipitate at the antigen site. Nuclei were counterstained with hematoxylin. Ten random microscopic fields per slide at a magnification of 400× were evaluated. To avoid errors in the observer’s field of view, the immunohistochemical results were interpreted by two experienced pathology instructors. Global ATG13 immunostaining were graded semi-quantitatively by multiplying the percentage of positively-stained cancer cells and the score of staining intensity as the final scores. The mean percentage of positively-stained cells was scored as 0-100%. The staining intensity was scored as follows: absent (0); weak (1); moderate (2); and strong (3). The multiplication of these parameters was used as the final staining score (0-300%). Patients were separated into low and high-expression groups according to the median value (148.75%) of the staining scores. For statistical evaluation, the tumor samples with a final score <148.75% were classified as low ATG13 expression and those with a score ≥148.75 as high ATG13 expression.

*Cell culture and transfection*. Two gastric cancer cell lines, denoted HGC27 (catalog no. TCHu22) and MKN45 (catalog no. TCHu280) from the Cell Bank of Chinese Academy of Sciences, were routinely cultured as described previously (21). A pre-designed validated siRNA against the sequence 5’-GCCATGTTTGCTCCCAAGAAT-3’ of human ATG13 was chemically synthesized from Takara Bio Inc. (Beijing, China). Cell transfection was performed using the Lipofectamine 2000 reagent (GE Healthcare Life Sciences, Little Chalfont, UK) following the manufacturer’s instructions. Cells were harvested 72 h post-transfection and used for subsequent experiments, which was repeated at least three times.

### Western blotting analysis

This assay has been described previously (21). Briefly, approximately 50 µg of total proteins extracted from cell lysates were separated on SDS-PAGE, transferred to PVDF membranes (EMD Millipore, Billerica, MA, USA) and incubated a ATG13 polyclonal antibody (Abcam) at a dilution of 1:500 overnight at 4 °C. Immunoblots were then probed with the secondary antibodies conjugated to horseradish peroxidase against rabbit IgG (Abcam), and signals were visualized using an enhanced chemiluminescence system (GE Healthcare Life Sciences, Chalfont, UK) in line with the manufacturer’s protocols. The blots were re-probed with a rabbit polyclonal antibody against GAPDH (Abcam) to confirm equal loading of different samples. The intensity of ATG13 was quantified using the Image J software.

### Cell proliferation and colony formation assays

The capacities of cell proliferation and colony formation were examined using a 3-(4, 5-dimethylthiazol-2-yl)-2, 5-diphenyl-2H-tetrazolium bromide (MTT) colorimetric assay and a monolayer colony formation assay respectively, according to the standard methods described before (21).

### Cell invasion assay

Cell invasive capacity was evaluated using a Matrigel invasion chamber assay described before (21). Briefly, 1×10^5^ cells per 500 μl of Dulbecco’s modified Eagle’s medium (Hyclone, Logan UT, USA) were seeded into the upper chamber of 24-well Matrigel-coated Transwell inserts (EMD Millipore). The lower chamber of the insert was filled with 750 μl of DMEM with 10% fetal bovine serum (Hyclone). After 24 h incubation, cells on the underside surfaces were fixed with 4% paraformaldehyde, stained with 2.5% crystal violet reagent (Sigma−Aldrich; Merck KGaA, Darmstadt, Germany) and counted in five random fields under the microscope.

### Statistical analysis

Statistical analyses were performed using the SPSS 17.0 statistical software package (SPSS Inc., Chicago, IL, USA). For bioinformatic processing of TCGA/GTEx/GEPIA data, the expression data are first log2 (FPKM/or TPM + 1) transformed for differential analysis and the log2FC was defined as median (Tumor)-median (Normal). The false discovery rate (FDR) using the Benjamini-Hochberg method was computed to correct for multiple hypothesis testing. Data from at least three independent cell culture experiments were presented as the mean ± standard deviation (SD). Continuous variables were compared using Student’s *t*-test or the Mann-Whitney U test, as appropriate based on data distribution. Categorical variables were analyzed using the Fisher’s exact test. The Kaplan-Meier method with a log-rank test was used to assess clinical outcome after surgery. Patients with missing data or loss of follow-up were not included in the above analyses. To identify independent prognostic factors, variables achieved significance in univariate analysis were included in a multivariate Cox proportional hazards regression model. The proportional hazards assumption was tested and confirmed. A two-tailed P-value <0.05 was considered statistically significant.

## Results

### Transcriptional expression profile of ATG13 in human cancers

**Figure 1:**
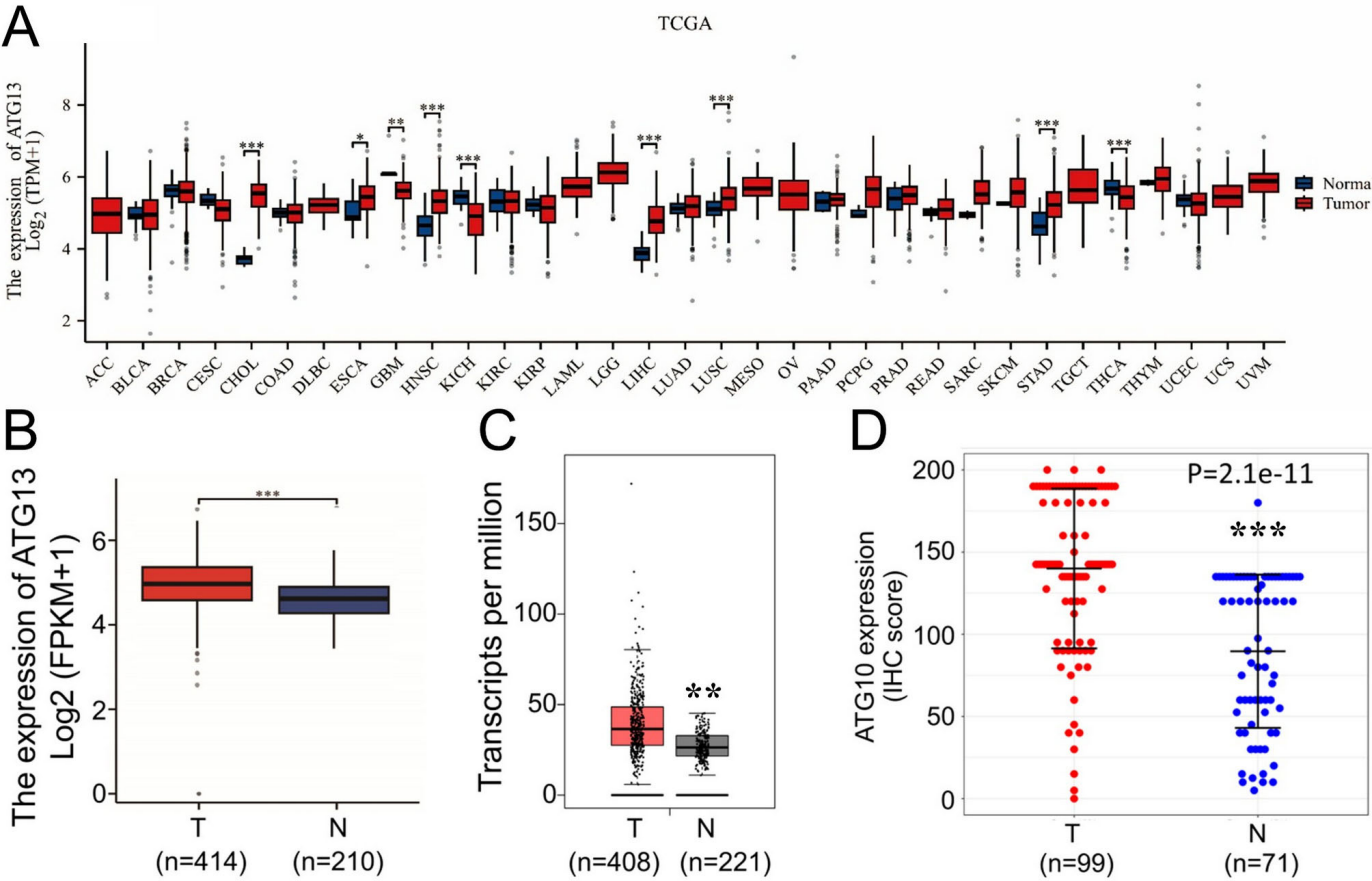
Transcription expression levels of ATG13 in diverse cancer types. **(A)** A graphic produced from the TCGA datasets shows ATG13 mRNA expression levels in cancer tissues along with the corresponding normal tissues across multiple cancer types. **(B)** ATG13 mRNA levels of in gastric cancer tissues and adjacent noncancerous tissues were compared using the GTEx datasets. **(C)** ATG13 mRNA levels were analyzed in STAD using the GEPIA database. **(D)** Immunohistochemical scores of ATG13 protein expression in STAD and paired adjacent noncancerous tissues. Unpaired Student’s *t*-tests with Welch’s correction were used to examine differential ATG13 protein expression in cancerous and adjacent noncancerous tissues. *P<0.05, **P<0.01, ***P<0.001. T, tumor tissues; N, normal tissues; STAD, stomach adenocarcinoma.

We set out to characterize the differences of ATG13 transcriptional levels between tumor and normal tissues across diverse cancer types by means of bioinformatics analysis. Using the date from TCGA, a graph was obtained to demonstrate the ATG13 mRNA expression profile in 33 cancer types including STAD. Among them, ATG13 mRNA expression was observed significantly higher in STAD tissues than in normal tissues (**Figure 1A**). Further analyses were conducted to demonstrate the differential ATG13 expression in gastric cancer tissues and their matching adjacent noncancerous tissues, using the data from GTEx. As expected, ATG13 expression was significantly increased in STAD tissues compared to the paired noncancerous tissues (**Figure 1B**). The above findings were supported by the observation from the GEPIA data base analysis (**Figure 1C**). In addition, the immunohistochemical analysis also confirmed upregulated ATG13 expression in STAD tissues compared to the matching adjacent noncancerous tissues (**Figure 1D**).

### Association between genomic alterations of ATG13 and patient survival

**Figure 2:**
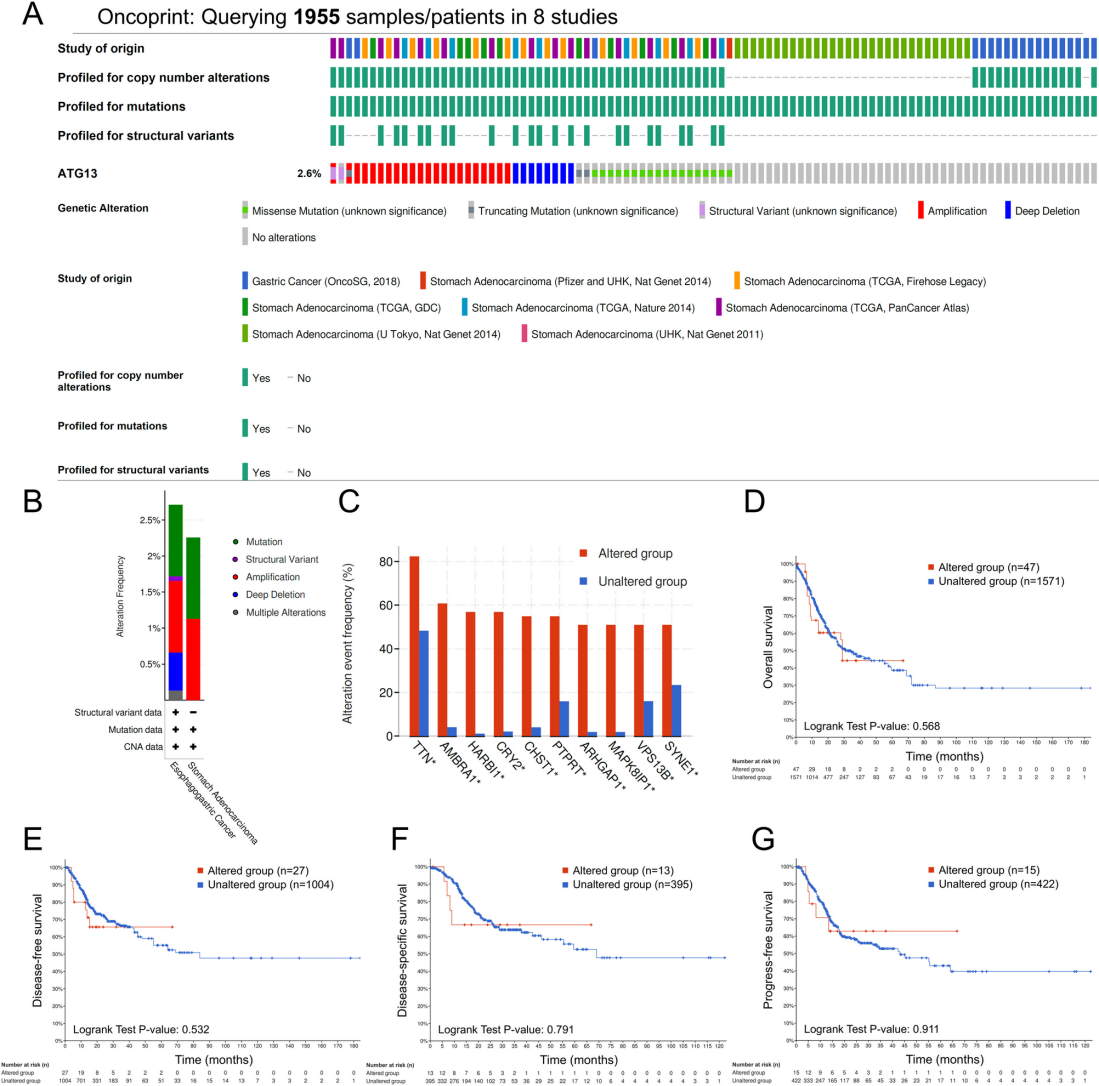
Association between genomic alterations of ATG13 and patient survival in gastric cancer via cBioPortal database analysis. **(A)** The proportion and distribution of samples with genomic alterations in ATG13 presented by OncoPrint in cBioPortal. The figure was cropped on the right side to exclude samples without alterations. **(B)** The alterations frequency of ATG13 in subtypes of gastric cancer. **(C)** Analyses of the top 10 genes with concomitant genomic alterations of ATG13. The impact of genomic alterations of ATG13 on **(D)** overall survival, **(E)** disease-free survival, **(F)** disease-specific survival and **(G)** progression-free survival in patients with gastric cancer. Survival curves were compared using the Kaplan-Meier plotter analysis with a log-rank test.

We next identified the prognostic association between genomic alterations of the ATG13 gene and patient survival using the cBioPortal online database. The genomic alteration rate for ATG13 was 2.6% in eight studies containing 1955 gastric cancer patients (**Figure 2A**). Mutation and amplification were observed to appear more frequently in both stomach adenocarcinoma and esophagogastric cancer (**Figure 2B**). Further correlation analyses revealed the top 10 most frequent co-altered genes whose genomic alterations were correlated with ATG13, including TTN, AMBRA1, HARBI1, CRY2, CHST1, PTPRT, ARHGAP1, MAPK8IP1, VPS13B and SYNE1 (**Figure 2C**). However, following analyses by Kaplan-Meier plot with a log-rank test, no significant association was found between genomic alterations of ATG13 and patient survival, as regarding OS, DFS, DSS or PFS (**Figures 2C–G**).

### ATG13 expression is an independent prognostic indicator in gastric cancer

**Figure 3:**
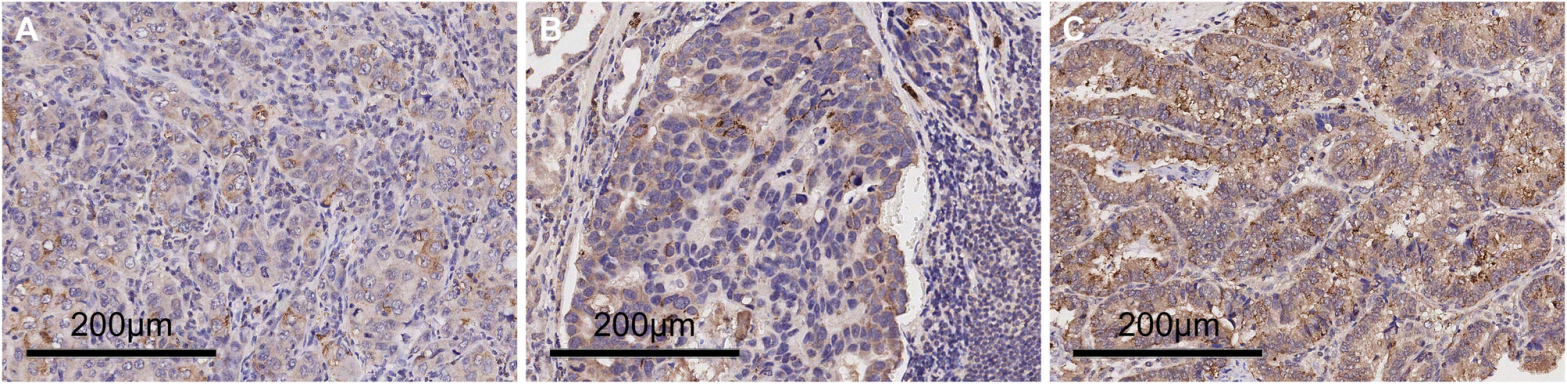
Representative immunohistochemical staining for ATG13 protein expression in gastric cancer tissues. **(A)** Weak staining; **(B)** Moderate staining; and **(C)** Strong staining. Original magnification, 200×.

**Figure 4:**
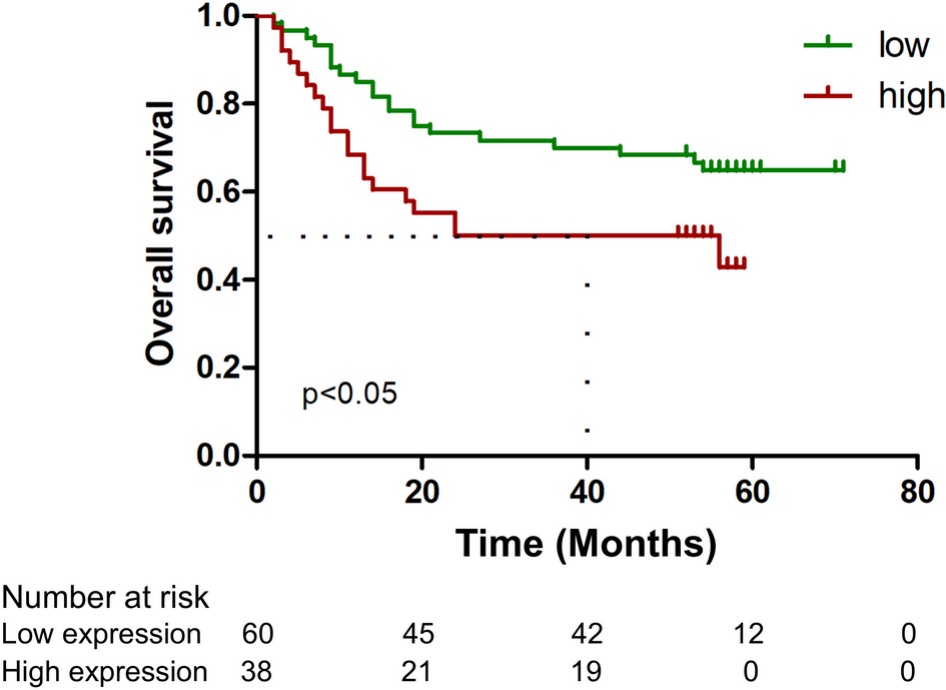
Kaplan-Meier curves comparing the survival outcomes according to the protein expression levels of ATG13. High ATG13 expression was significantly correlated with a worse OS rate in patients with gastric cancer. Survival curves were generated using the Kaplan-Meier method and compared with a log-rank test.

We then conducted immunohistochemistry to determine ATG13 protein expression using one tissue microarray chip containing total 99 primary STAD specimens. We found that high ATG13 immunostaining was observed in the cytoplasm of cancer cells in 39.4% (39/99) of the cancer samples tested (**Figure 3**). We found that high ATG13 expression was significantly associated with larger tumor size (**Table 1**). Kaplan-Meier survival analysis revealed that patients with high ATG13 expression had a worse OS than those with low ATG13 expression (**Figure 4**). Upon univariate analysis, ATG13 expression, T stage (invasion depth), N stage (lymph node metastasis), and M stage (distant metastasis) were detected to be responsible for an unfavorable OS (**Table 2**). Following adjusting the prognostic variables from the univariate analysis, ATG13 expression, N stage and M stage kept independent values in the multivariate analysis (**Table 2**).

**Table 1:** Association between ATG13 expression and clinicopathological characteristics in patients with gastric cancer.

Variables	No. of patients	ATG13 expression		*P* value
		Low, *n* (%)	High, *n* (%)	
Age (years)				
≤66	50	33 (66.0)	17 (34.0)	0.307
>66	49	27 (55.1)	22 (44.9)	
Sex				
Male	30	18 (60.0)	12 (40.0)	1.000
Female	69	42 (60.9)	27 (39.1)	
Histological grade				
I-II	59	34 (57.6)	25 (42.4)	0.532
III	40	26 (65.0)	14 (35.0)	
Tumor size (cm)				
≤6	16	15 (93.8)	1 (6.2)	0.002
>6	81	43 (53.1)	38 (46.9)	
NA	2			
Clinical stage (pTNM)				
I-II	38	22 (57.9)	16 (42.1)	0.833
III-IV	60	37 (61.7)	23 (38.3)	
NA	1			
T Stage				
T1-T3	67	42 (62.9)	25 (37.3)	0.510
T4	31	17 (54.8)	14 (45.2)	
NA	1			
N Stage				
N0	24	13 (54.2)	11 (45.8)	0.480
N1-N3	75	47 (62.7)	28 (37.3)	
M Stage				
M0	96	57 (59.4)	39 (40.6)	0.276
M1	3	3 (100.0)	0 (0.0)	

NA, not available.

**Table 2:** Univariate and multivariate analyses for OS in patients with gastric cancer.

Variables	Univariate analysis		Multivariate analysis	
	HR (95% CI)	*P* value	HR (95% CI)	*P* value
ATG13 expression	1.90 (1.03-3.52)	0.040	2.27 (1.18-4.34)	0.014
T stage	2.37 (1.28-4.40)	0.006	1.53 (0.80-2.94)	0.198
N stage	3.80 (1.35-10.69)	0.011	3.52 (1.21-10.20)	0.021
M stage	6.98 (2.01-24.19)	0.002	6.82 (1.81-25.72)	0.005

HR, hazard ratio; CI, conﬁdence interval.

### Silencing ATG13 expression inhibits gastric cancer cell growth and metastasis

**Figure 5:**
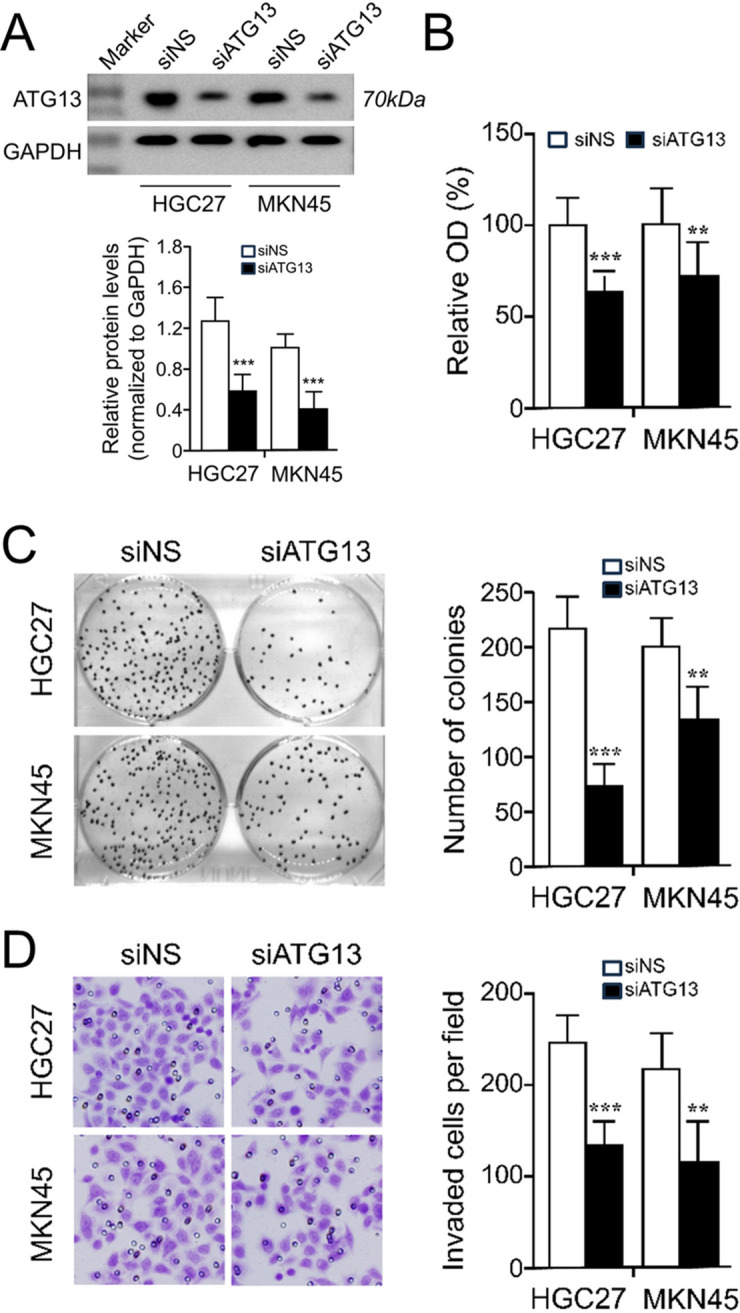
Silencing ATG13 expression inhibits gastric cancer cell growth and metastasis. Cells were pretreated with or without ATG13 siRNA for 72 h and then subjected to the following assays. **(A)** Knockdown of ATG13 in HGC27 and MKN45 cells as indicated western blot. *Upper*, representative WB result; *lower*, quantitative analysis from three experiments normalized to GAPDH. Knockdown of ATG13 repressed the growth of HGC27 and MKN45 cells as examined by MTT assay **(B)** and colony formation assay **(C)**. **(D)** Knockdown of ATG13 repressed the invasiveness of HGC27 and MKN45 cells using a Matrigel invasion chamber assay. Representative images are shown in the left panel and quantitative analysis are displayed in the right panel. Data are presented as the mean ± SD of three independent experiments. Statistical analysis was determined by Mann-Whitney U test. **P<0.01, ***P<0.001, as compared to the control group. siNS, non-specific control siRNA.

We further investigated the biological functions of silencing ATG13 expression in gastric cancer cells. The efficiency of ATG13 silencing using a pre-design validated siRNA was examined by western blot analysis (**Figure 5A**). We found that HGC27 and MKN45 cells with ATG13 knockdown exhibited a significant decrease in cell proliferation and colony formation compared to their control cells transfected with non-specific control siRNAs (**Figures 5B, C**). Additionally, the impact of ATG13 knockdown on cell invasiveness was evaluated using a Matrigel assay. By doing so, we observed that ATG13 knockdown dramatically decrease the number of invaded cells in both cell lines (**Figure 5D**). These findings suggest that silencing ATG13 expression suppresses the growth and metastasis of gastric cancer cells.

## Discussion

An effective and valuable prognostic biomarker is of urgent need to improve individualized therapy for gastric cancer patients. In the present study, we examine the expression as well as prognosis significance of ATG13 expression in gastric cancer. Our findings suggest that ATG13 expression is frequently upregulated in gastric cancer tissues and may serve as promising prognostic indicators for gastric cancer patients.

The biological functions of ATG13 focusing on autophagy has received much attention recently, for the reason that it is a critical participant for autophagy initiation. It has reported that ATG13/ULK1 signaling is involved in the autophagy induction among multiple cancer types (22–25). Regarding gastric cancer, it has been found that THADA inhibits autophagy and make gastric cancer cells more sensitive to 5-FU via downregulating ATG13 expression and activating the PI3K/AKT/mTOR signaling pathway (26). All these above findings suggest that ATG13 might be employed as a promising potential target for cancer tailor-made therapy, although its effect on different cancer types remains controversial. However, the clinicopathological correlation and prognostic implication of ATG13 expression in cancers has not been reported as yet. The present study thus examined the expression profile of ATG13 in gastric cancer and its association with clinicopathological parameters and patient survival. High ATG13 expression was observed to be associated with tumor size and an unfavorable OS. We thus conclude that ATG13 protein expression may act as an independent prognostic factor for survival prediction in gastric cancer.

Based on the above findings, we subsequently performed several basic biological functional experiments to explore the pilot nature of ATG13 expression on malignant properties of gastric cancer cells. As expected, knockdown of ATG13 expression significantly suppressed the malignant phenotypes of gastric cancer such as cell proliferation and metastasis. These results are consistent with the previous report in breast cancer (12), and also supports our observation in immunohistochemistry by which increased CDK10 expression was associated with an unfavorable OS in patients with gastric cancer. Our data suggest that dysregulated ATG13 expression might influence and consequently be related with aggressive behavior of gastric cancer cells.

There are several limitations to be addressed as regarding the current study. Because the current study performed integrative bioinformatics analyses using a set of online databases, certain search parameters were not available. The limited sample size and restricted follow-up period in the immunohistochemical analysis demands enlarged sample cohort with integral information in our future work. Meanwhile, additional analyses also should be carried out, e.g., application of a RCT analysis, and validation of ATG13 expression via an independent cohort or external dataset and a more precise multivariate prognostic model with C-index and internal validation. More importantly, because this manuscript is generally a prognostic/exploratory biomarker analysis, we just preliminarily performed several additional basic cellular experiments to explore the impact of dysregulated ATG13 expression on cancer cell phenotypes. The present study should be a prognostic/exploratory biomarker analysis rather than a mechanistic investigation. The nature of the *in vitro* works might be only a pilot proof-of-principle study. Thus, the biological functions and molecular mechanism of ATG13 in gastric cancer require to be further validated under well-controlled conditions in cell and/or animal models, e.g., utilization of multiple siRNAs and rescue assays to confirm specificity, and application of autophagy flux assays (e.g., LC3-II, p62, lysosomal inhibition) for direct evidence of autophagy modulation.

In summary, we systematically investigated the expression profile of ATG13 and its corresponding prognostic implications in gastric cancer. Although the present study has some limitations to generalize the results (e.g., sample size and restricted follow-up periods), we provided the evidence of ATG13 as a prognostic biomarker in gastric cancer, Nonetheless, the underlying molecular mechanisms of ATG13 in gastric cancer should be validated by performing further *in vitro* and *in vivo* studies to strengthen the biological role of ATG13 in our future work.

## Data Availability

The data presented in the study are deposited in the public repository Zenodo with DOI: 10.5281/zenodo.17875112.
