# Supplementary figures and images for: The clinical significance and prognostic implication of autophagy-related gene 13 in human gastric cancer
Source: Front Oncol. 2026 Feb 6;16:1729996. doi: 10.3389/fonc.2026.1729996 (PMC12920215; doi:10.3389/fonc.2026.1729996)

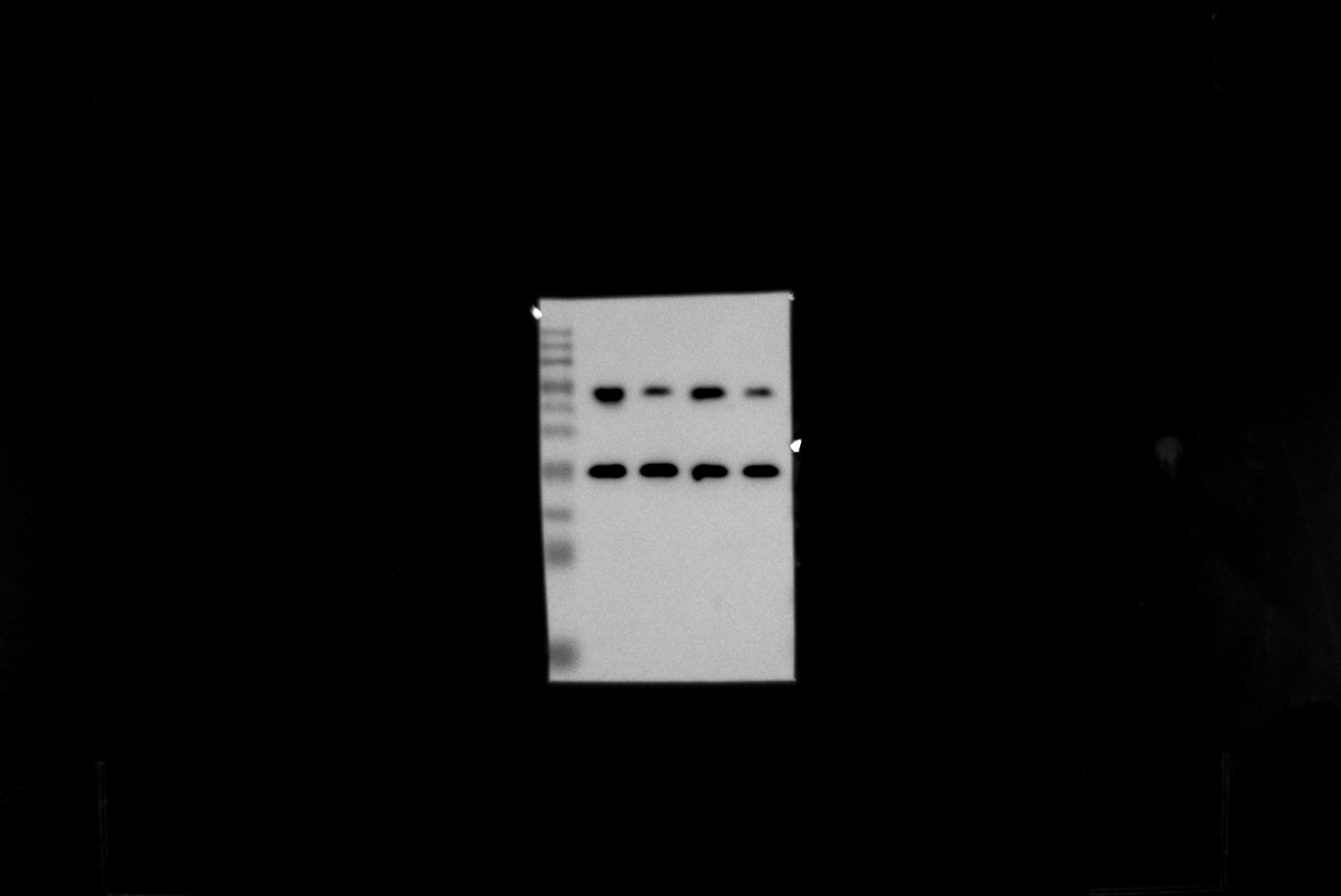

Supplement: Supplementary file 1 [file Presentation1.zip › FULL/70kd合并图.tif]

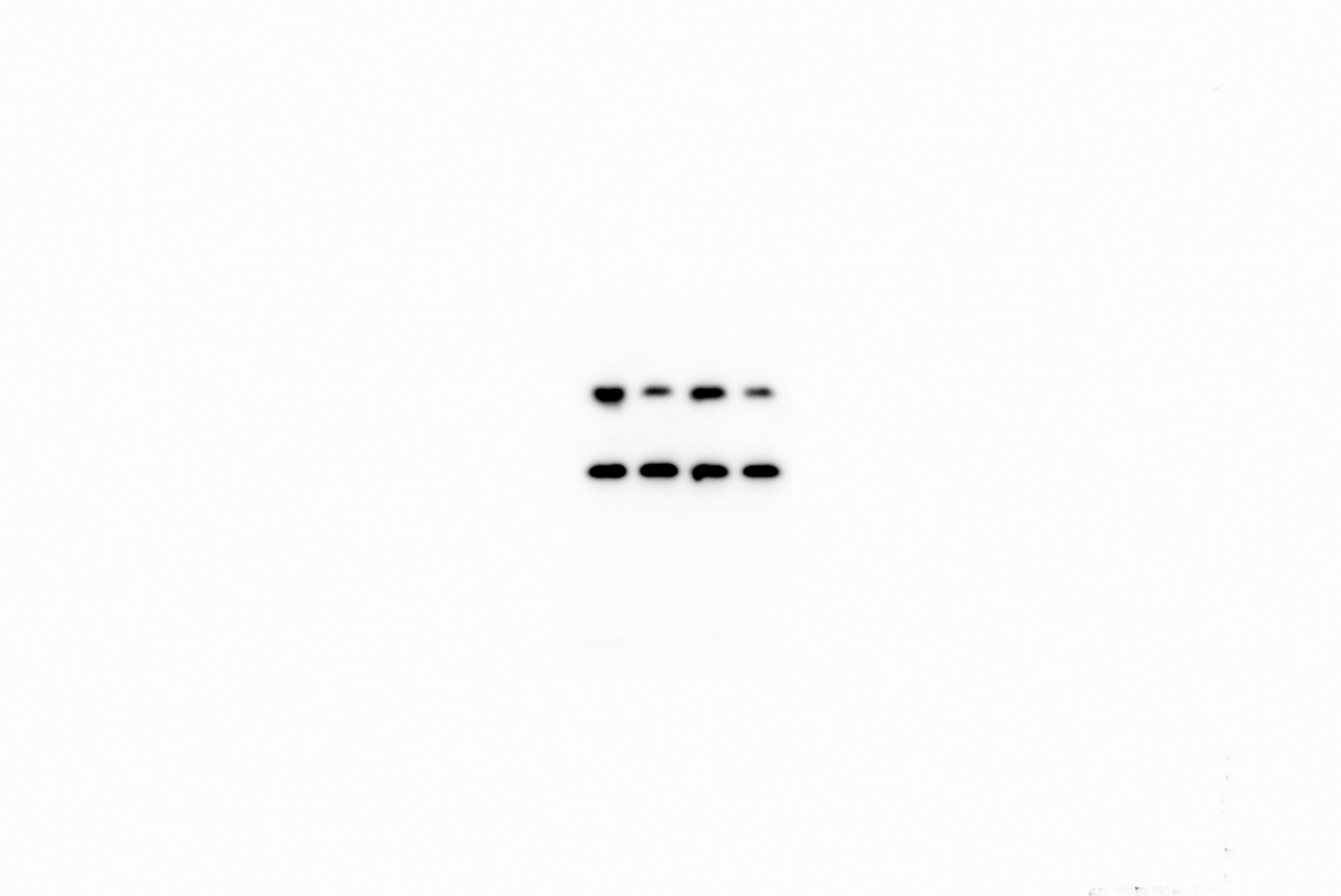

Supplement: Supplementary file 1 [file Presentation1.zip › FULL/70kd条带图.tif]

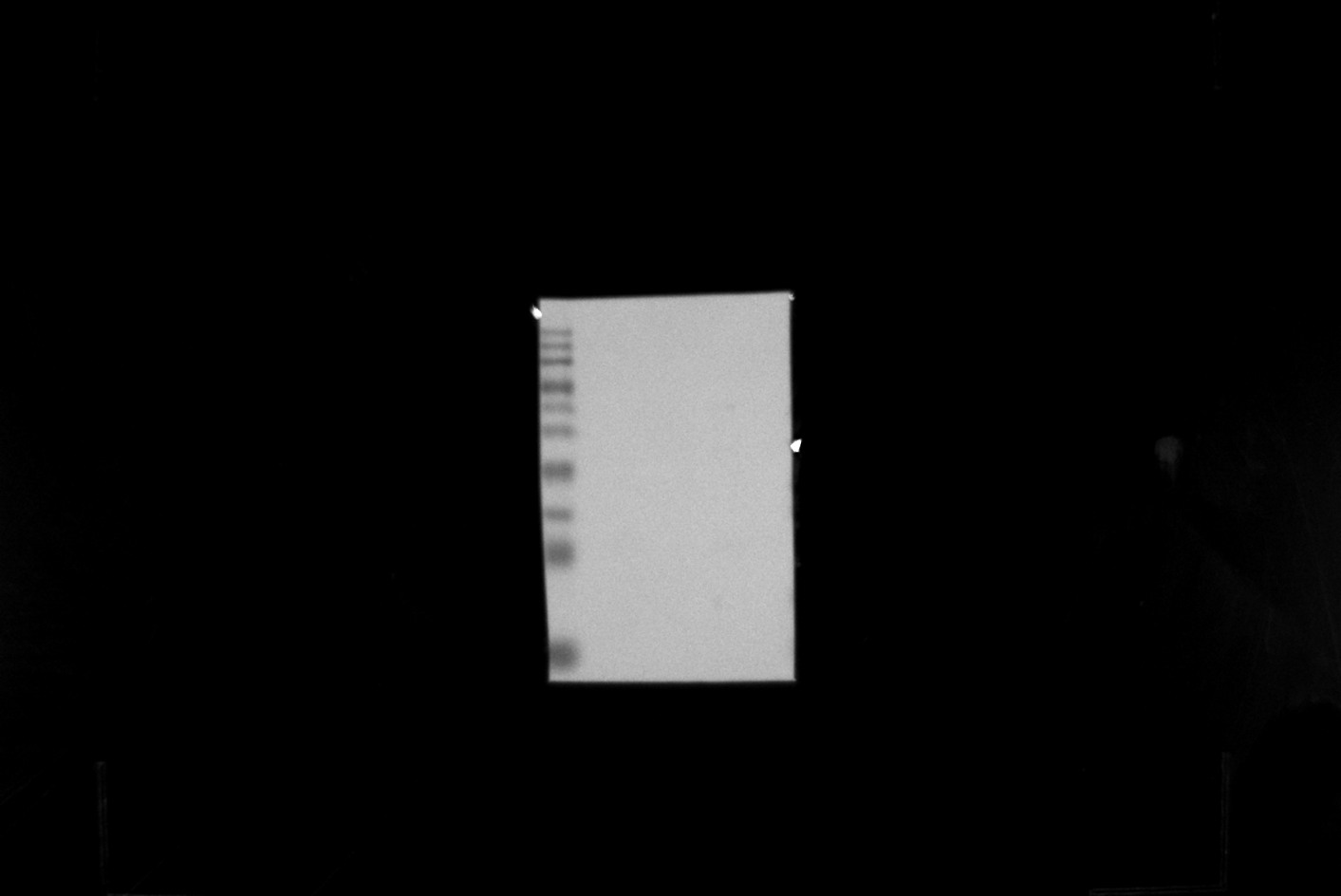

Supplement: Supplementary file 1 [file Presentation1.zip › FULL/70kd白光图.tif]
